# Evaluating the (cost-)effectiveness of guided and unguided Internet-based self-help for problematic alcohol use in employees - a three arm randomized controlled trial

**DOI:** 10.1186/s12889-015-2375-0

**Published:** 2015-10-12

**Authors:** Leif Boß, Dirk Lehr, Matthias Berking, Heleen Riper, Michael Patrick Schaub, David Daniel Ebert

**Affiliations:** Division of Health Training Online, Innovation Incubator, Leuphana University Lueneburg, Rotenbleicherweg 67, D-21335 Lueneburg, Germany; Department of Clinical Psychology and Psychotherapy, Friedrich-Alexander University Erlangen Nuremberg, Nägelsbachstraße 25a, D-90503 Erlangen, Germany; Swiss Research Institute for Public Health and Addiction, University of Zurich, Konradstrasse 32, CH-8031 Zürich, Switzerland

**Keywords:** Internet intervention, Alcohol, Work-related stress, Occupational health, Cost-effectiveness, Self-help, Problematic alcohol use, Alcohol use disorders, Randomized controlled trial

## Abstract

**Background:**

Problematic alcohol consumption is associated with a high disease burden for affected individuals and has a detrimental impact on companies and society due to direct and indirect health costs. This protocol describes a study design to evaluate the (cost)-effectiveness of a guided and unguided Internet-based self-help intervention for employees called “GET.ON Clever weniger trinken” (be smart – drink less) compared to a waiting list control group.

**Methods:**

In a three-arm randomized controlled trial, 528 German adults who are currently members of the workforce will be recruited by occupational health departments of major health insurance companies. Employees aged 18 and older displaying problematic drinking patterns (>21/14 drinks per week and an AUDIT score > 8/6 for men/women) will be randomly assigned to one of three following study conditions: 1. unguided web-based self-help for problematic drinking, 2. adherence-focused guided self-help, and 3. waiting list control. Self-report data will be collected at baseline (T1), 6 weeks (T2), and 6 months (T3) after randomization. The primary outcome will be the reduction of alcohol standard units during the 7 days prior to T2, using the Timeline Followback method. Cost-effectiveness analyses to determine direct and indirect costs will be conducted from the perspectives of employers and the society. Data will be analyzed on an intention-to-treat basis and per protocol.

**Discussion:**

There is a need to identify effective low-threshold solutions to improve ill-health and reduce the negative economic consequences due to problematic alcohol drinking in workforces. If the proposed web-based intervention proves both to be efficacious and cost-effective, it may be a useful tool to increase utilization rates of interventions for problematic drinking in occupational settings.

**Trial Registration:**

German Register of Clinical Studies (DRKS): DRKS00006105, date of registration: 2014-07-07.

## Background

### Problematic alcohol use - a global health problem

Problematic alcohol consumption is associated with a high burden of disease [1, 2]. Alcohol use disorders (AUDs) are projected to become the fourth leading cause of disability in high-income countries by 2030 [3]. The 12-month prevalence for alcohol dependence in the U.S. population is estimated to be 7 % [4] and in the German population 3.4 % [5]. AUDs are also linked to mental health problem domains, such as mood and anxiety disorders [6], work stress [7] and are associated with an increased risk for premature mortality [8].

However, the proportion of people with problematic drinking patterns that exceed the low-risk threshold but do not result in an AUD is even higher and might be more suitable to illustrate the actual dimension of the health problem [9]. Prevalence rates of such patterns vary considerably because there is no consensus when problematic drinking begins [10, 11]. For example, in 2013, 24.6 % of the U.S. population reported binge drinking, i.e., having five or more drinks in one occasion within the last month [4]. In the German population, 14.2 % of men and women drink more than 24 and 12 g of alcohol per day, respectively [5], and 33.6 % engage in hazardous drinking as defined by the short form Alcohol Use Disorders Identification Test [12].

In the present study, problematic drinking is alcohol consumption that is likely to lead to physical or psychosocial harm and will be defined based on the recommendations of the World Health Organization [13]. According to this, people engaging in problematic drinking consume more than 14 (women) or 21 (men) standard units of alcohol per week. Problematic alcohol consumption is associated with considerable costs due to impaired productivity and absence from work [14, 15]. Total alcohol-attributable costs per person range between $358 and $837 in high-income countries [2]. Indirect costs such as those due to productivity losses have shown to be the predominant cost-category with an average 72.1 % of all alcohol-attributable costs in high-income countries [2].

There are good reasons for offering services that help to reduce alcohol consumption in occupational settings. Workplaces offer a high potential for delivering alcohol prevention by reaching a high proportion of the target group [16, 17]. As there are correlations between alcohol drinking, absence from work, and related costs [18], occupational prevention programs may help to reduce impaired productivity due to, for example, alcohol-related absenteeism and presenteeism [19]. Moreover, Siegrist and Roedel [20] found evidence from prospective studies that work-related stress is a risk factor for problematic alcohol consumption. Based on a social learning paradigm, people may use alcohol as an alternative mechanism to cope with stressful situations (e.g. work stress) [21]. Such situations may include difficulties to relax from work or to cope with negative emotions. According to social environment models, peer pressure and the omnipresent availability of alcohol may add to the risk of increase drinking in those situations. Reducing these risk factors by providing exercises of emotional coping as a major part of an intervention might be beneficial.

### Existing treatments at the workplace

Different approaches have been tested in occupational settings, for example, education programs, personal counseling, individual feedback, brief mail-out interventions, and management training [22]. However, effects of these interventions are mixed, and many studies lack sufficient methodological quality [22, 23]. Traditional occupational interventions are typically offered in large businesses that have an employee assistance program or other health-promoting plans. Thus, especially people in smaller businesses are less likely to have access to these kinds of prevention programs [16]. Another barrier for implementing health interventions at the workplace may be low participation rates [23]. Reasons for these low rates include a preference for self-helping attempts [24] and a fear of stigmatization [25, 26].

### Potential of web-based interventions

Using the Internet to provide brief self-help interventions may help to overcome some of the barriers for implementing traditional occupational health programs. People can access the intervention at any time and at any place without disclosing their identity [16]. Other advantages include the fact that participants can work at their own pace and review materials as often as they want. In addition, such interventions possibly reach affected people earlier than traditional health services, thereby preventing the onset of more severe health problems [27].

### Efficacy of web-based interventions for problematic alcohol use

In recent years, studies on web-based interventions for alcohol reduction have been on the rise [28, 29]. Meta-analyses have revealed effect sizes of these kinds of interventions for reducing weekly alcohol units ranging from g = 0.2 [28] to d = 0.4 [29].

### Web-based occupational health interventions for problematic alcohol use

There have only been a few studies on web-based alcohol interventions in occupational settings. For example, employees from an U.S. technology company participated in a web-based health promotion program designed for universal prevention of depression, anxiety, and problematic substance use [30]. This study showed that employees participating in this program were slightly more willing to change their drinking behavior compared to those in the control group. However, data of the total amount of drinking was not reported. Doumas and Hannah [31] tested a brief website that provides personalized normative feedback (PNF) on drinking to young employees in the 18-24-year age group. The study group found small effects on reductions of weekend drinking (d = 0.3), peak consumption (d = 0.3), and intoxication (d = 0.2) compared to a control group. Pemberton and his co-authors [32] tested a brief web-based intervention for high-risk drinkers and a web-based universal prevention program including PNF, motivational interviewing elements, and skills for behavioral change in military personnel. They found small effects for the intervention for high-risk drinkers on average drinks per drinking occasion, frequent heavy episodic drinking status, and estimated peak blood alcohol concentration (all about d = 0.1 compared to a control group). In contrast, the effects of the prevention program were not significant. In a more recent study, Khadjesari and colleagues [33] analyzed the effects of an online screening and PNF in a workforce in the UK, but did not find improvements with regard to drinking behavior. In the long term, after 6 months, none of these interventions showed significant effects.

However, all described interventions were tested in very specific populations, for example, military personnel [32], adolescents [31], individual companies [33], thus, it is questionable, if findings can be generalized to other workforce populations. Moreover, to the best of our knowledge, none of these studies included an economic evaluation. All of the interventions mentioned above are based on a self-help paradigm. However, unguided web-based interventions, that means those without any human support (i.e., pure self-help), have been found to be less effective than guided interventions for depression and social phobia [34]. With regard to interventions for problematic alcohol consumption, the picture is less clear. In a recent meta-analysis, Riper and her co-authors [28] did not find differences in terms of efficacy between guided and unguided interventions across different studies, but the number of trials with guidance was very small (*n* = 5). Thus, there is a need to explore the (cost-)effectiveness of web-based interventions for reducing problematic alcohol consumption with and without guidance in the same study.

## Aims of the study

The scope of this study is to evaluate the (cost-)effectiveness of a newly developed web-based cognitive-behavioral self-help intervention called GET.ON Clever weniger trinken (CWT) for employees with problematic alcohol consumption. The study has the following aims: 1) to assess the effectiveness of self-help CWT for reducing alcohol consumption compared to a control group, 2) to assess the effectiveness of CWT with adherence-focused guidance compared to a control group, 3) to assess the incremental cost-effectiveness ratio (ICER), that is the ratio between costs and clinical outcome, of guided and unguided CWT compared to a control group. We expect both intervention groups to be superior compared to the control group in terms of alcohol consumption reduction from baseline to the post-assessment. We hypothesize guided and unguided self-help CWT both to be more cost-effective compared to the control group at the 6-month follow-up assessment. As a secondary aim we explore the differences of additional professional support, i.e., adherence-focused guidance [35].

## Methods

### Study design

**Fig. 1:**
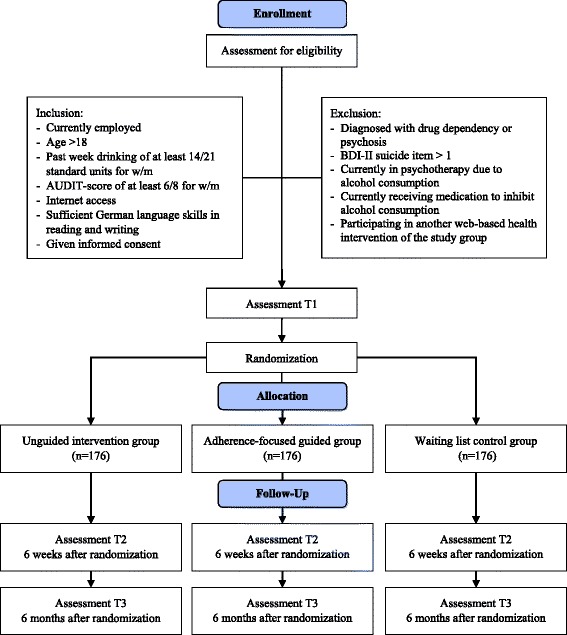
Study flow

A three-arm randomized controlled trial (RCT) will be conducted to evaluate the web-based intervention CWT with and without guidance compared to a waiting list control group (WLC). Assessments will take place before the allocation to the study conditions (T1), 6 weeks (T2), and 6 months (T3) after the allocation (Fig. 1). All procedures involved in the study will be consistent with the generally accepted standards of ethical practice approved by the University of Lueneburg (Germany) ethics committee (No. Boss201404_OT). The trial is registered in the German clinical trials register DRKS00006105.

### Participants & procedure

#### Inclusion and exclusion criteria

We include (a) working people, (b) who are above the age of 18, (c) who report drinking of at least 14/21 (women/men) standard units per week, (d) who have a score of at least 6/8 for women/men on the Alcohol Use Disorders Identification Test (AUDIT) [36], (e) who have Internet access, (f) who have sufficient German language reading and writing skills (self-reported), and (g) who are willing to give informed consent. We exclude subjects (a) who indicate that they have been diagnosed with psychosis or a drug dependency in the past, (b) who show a notable suicidal risk as indicated by a score greater than 1 on BDI [37] Item 9 (“I feel I would be better off dead”), (c) who have received medication or have begun psychotherapy to treat their problematic alcohol consumption, and (d) who are participating in another study on online-health training of our study group at the same time.

#### Recruitment

Participants will be recruited nationwide from the German-speaking population. The recruitment process is scheduled from autumn 2014 to autumn 2015 and will be conducted by several health insurance companies (BARMER GEK, KKH, BKK). The insurance companies advertise the study in their member-journals that will be sent to all of their insurants and they promote the study participation on their websites. Nevertheless, participation is not limited to the insurants of these companies and is not restricted to specific industrial sectors or occupational groups. An open access website (http://www.geton-training.de/alkohol) provides information on the intervention and study conditions. Potential participants sign up by providing an email address and name or pseudonym on the website.

#### Assessment of eligibility and randomization

The trial will be open to all people who meet criteria listed above. After registering, applicants receive an email with detailed information about the study procedures. Then, they will be informed that they can withdraw from the intervention and/or study at any time without any negative consequences. Applicants who continue to participate in the study will be asked to complete an online screening questionnaire. They must fulfill all criteria of inclusion and none of the exclusion criteria, have to complete the baseline assessment (T1), and return the informed consent form to participate in the study.

Eligible applicants will be randomly allocated in a 1/1/1 ratio to one of the three trial arms: adherence-focused guided CWT, unguided CWT, or WLC. Randomization will take place at an individual level. The allocation will be performed by an independent researcher not otherwise involved in the study, using an automated computer-based random integer generator (randomisation.eu). During the randomization process, allocation will be concealed from participants, researchers involved in recruitment, and eCoaches. After being informed about the outcome of the randomization, participants in the two intervention groups will receive immediate access to the training. All data is collected using a secure web-based assessment system (AES, 256-bit encrypted).

### Intervention

**Fig. 2:**
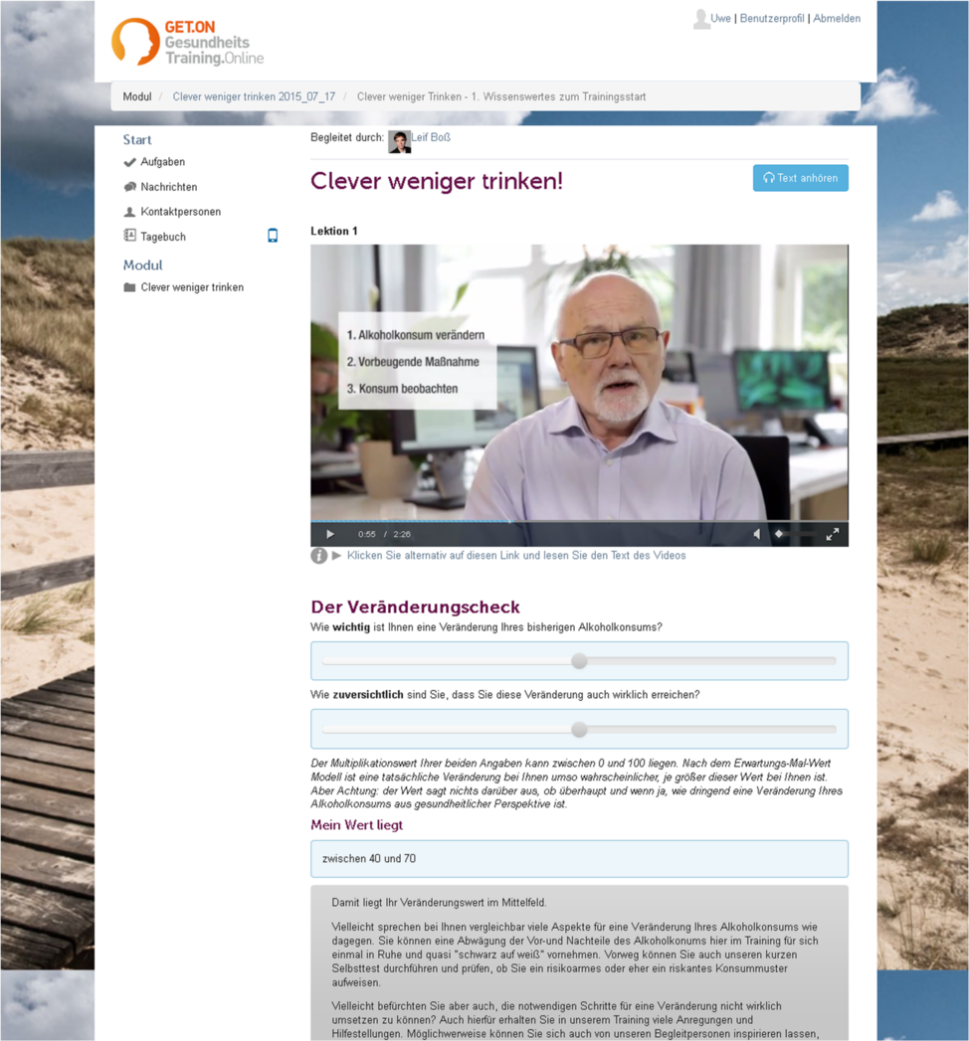
Video that introduces training goals

**Fig. 3:**
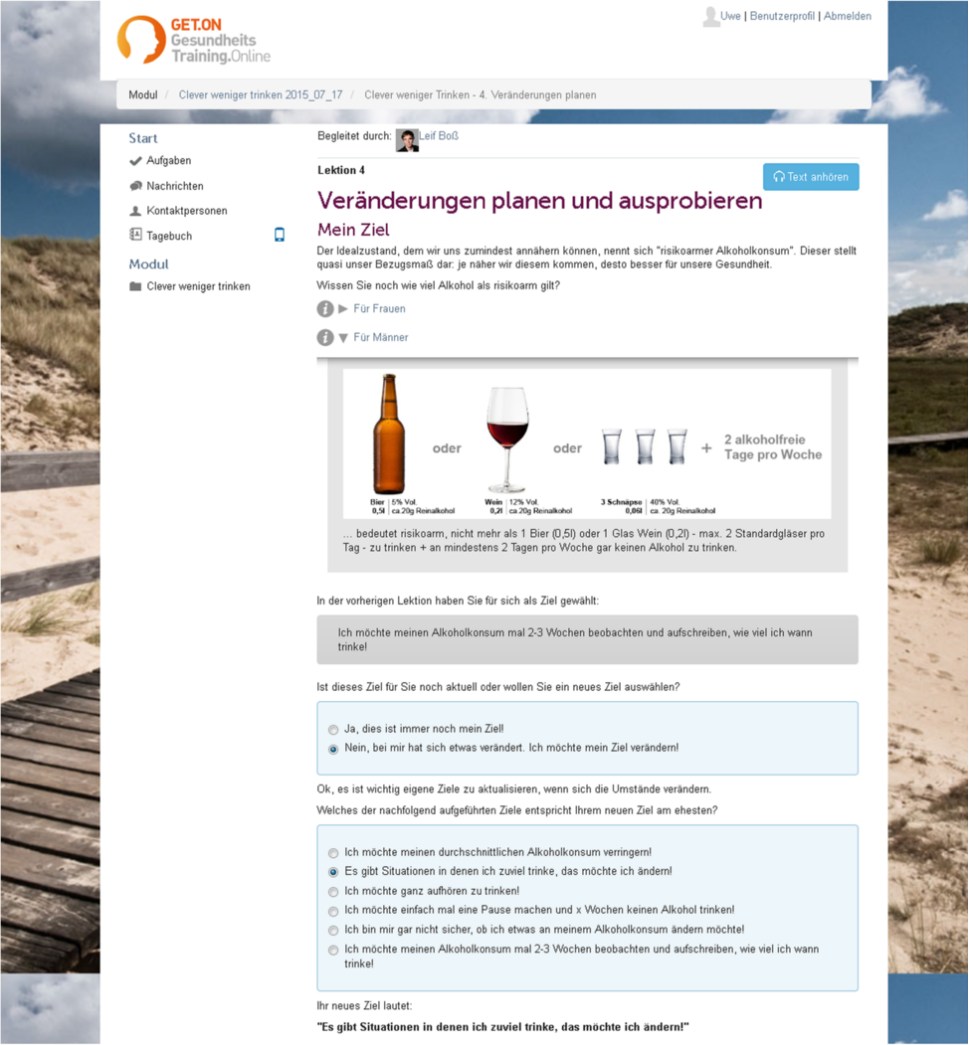
Example of adaptive content

**Fig. 4:**
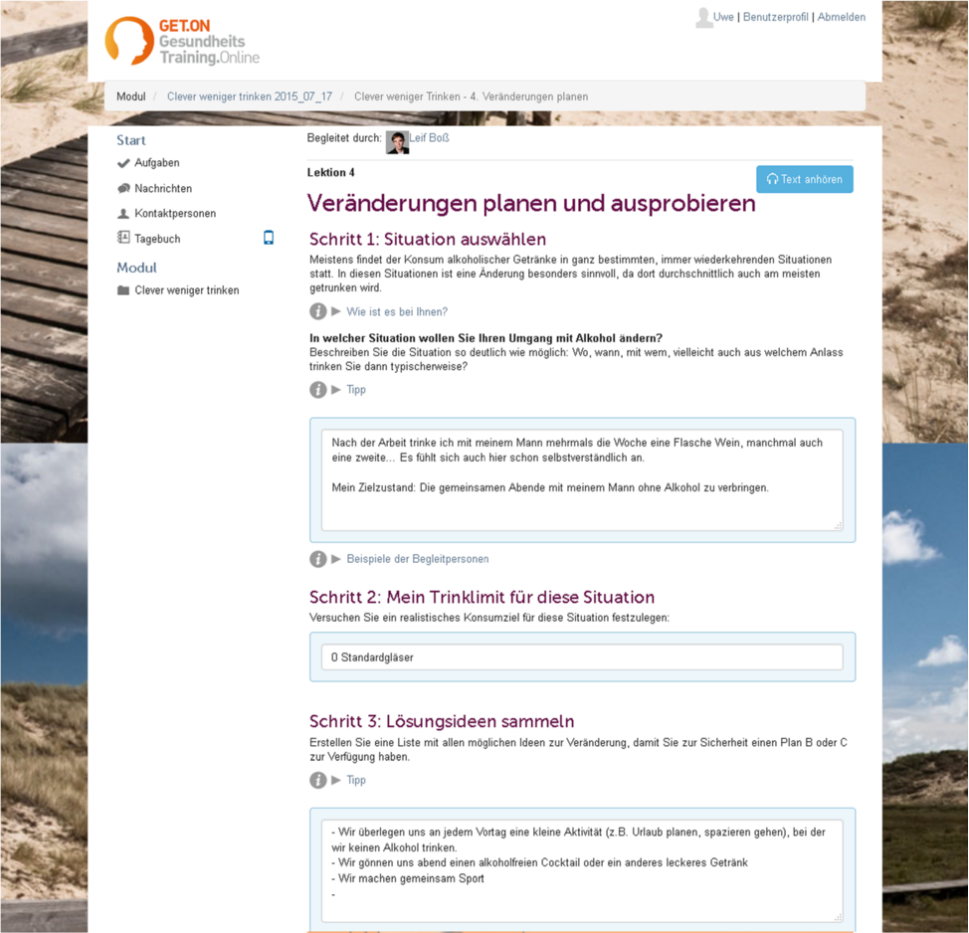
Example of a writing exercise for behavioral change planning

The web-based intervention CWT comprises five modules (Table 1). Each includes general information, illustrative examples, interactive exercises, quizzes, audio and video files, and downloadable work sheets (Figs. 2, 3, 4). The intervention combines different examples of good clinical practice in alcohol treatment [38], tools to control drinking behavior [39], and an emotion regulation training [40]. The combination of these elements is meant to initiate and promote the processes of change that allow participants to move from one stage of behavioral change to another, as defined by the Transtheoretical Model of Health Behavior Change [41].

**Table 1:** Content of the web-based training GET.ON CWT

Module	Intervention content
1	Psychoeducation
	Personalized normative feedback
	Motivational interviewing
2	Planning of behavioral change
3	Maintenance of behavioral change
	Emotion regulation
	Behavioral activation
4	Maintenance of behavioral change
	Emotion regulation
5	Planning for the future

Participants are advised to complete each module within one week. The Module 1 includes three major sections: section a) provides an overview of the training content, an explanatory model of conditions that may lead to increased consumption of alcohol (e.g. the wish to relax after work or some kind of peer pressure), and an explanation of alcohol standard units and why this measure is useful to monitor alcohol consumption. Section b) consists of personalized normative feedback. By completing a short self-assessment, participants identify their own drinking patterns in comparison to normative drinking guidelines. Participants who show a drinking pattern of high risk for alcohol dependency receive information about health services they should use in addition to the online intervention. Participants belonging to a high-risk group (e.g. pregnant participants) are advised to abstain from alcohol. This kind of a normative feedback element as stand-alone-intervention has been shown to effectively reduce drinking [42]. It aims to enable participants to reconsider their drinking habits by comparing their own alcohol consumption to that of peers and health norms [43]. In section c) different exercises are presented in a non-directive style based on motivational interviewing principles [44] that are meant to elicit behavioral change. The participants reflect on advantages and disadvantages of their drinking, think of reasons for change, and determine a personal goal (e.g. to reduce alcohol consumption in specific situations, to become abstinent, or to just monitor drinking habits).

An additional tool of the training program is an online-diary, which participants can use to record how much they drank on the previous day and to set a personal limit for the next day. At the beginning of each subsequent module, participants reflect on their drinking on the previous days. The diary is accessible via Internet or smartphone.

The core element in Module 2 is a four-step plan to control alcohol consumption in specific situations. It is theoretically based on the Health Action Process Approach (HAPA) [45] and elements of the Problem Solving Therapy (PST) [46]. The plan consists of the following steps: 1) Participants choose a typical situation in which it is hard for them to abstain from alcohol, 2) determine a drinking limit for this situation, 3) explore possible solutions for behavioral change in this kind of situation, and 4) describe in detail how to put their solution into practice. At the end of Modules 2 to 5, participants receive additional information and techniques they can use to achieve their goals. This optional toolbox contains information on the following topics: how to refuse alcohol in social contexts, how to control situations in which alcohol is easily available (stimulus control), how to change drinking habits, and how to relax after work without drinking (relaxation techniques).

In Module 3, participants reflect on their first efforts of controlling alcohol, adopt or adapt a plan for behavioral change, or develop a new plan in response to another problematic drinking situation. Participants are then introduced to the nature of different emotions and how these are linked to alcohol consumption. There is evidence for the detrimental impact of negative emotional states on maladaptive drinking [47, 48]. Furthermore, participants start to learn evoking positive emotions without using alcohol, for example, by planning enjoyable activities. This planning process can be continued throughout the other modules.

Module 4 comprises techniques of emotional regulation to cope with negative affective situations. The core exercise is to accept and tolerate negative emotional states based on the Affect Regulation Training (ART) [40, 49]. Acquiring these competencies may help individuals to improve their drinking habits [50]. Participants begin this exercise by recognizing a situation in the past when they had to struggle with their emotions. Then, they reflect on the usefulness and positive aspects of the unwanted (negative) emotions and develop strategies for coping with them in this kind of situation (i.e., by accepting the current emotional state). At the end of the exercise, they are reminded that they are able to bear the acute emotion and that this state will pass.

Finally, in Module 5, participants think of their progress and describe how they can continue to improve. They define an alcohol limit for the future and choose techniques that appeared to be useful to stay within the limit that they have set for themselves.

### Study conditions

#### Unguided CWT

Participants of the unguided intervention group will communicate with the team organizing the study during the study period but will not be supported by an eCoach. In the case of any technical problems, they can contact support via email.

#### Adherence-focused guided CWT

Participants of the guided intervention group will be supported by an eCoach. Guidance is mainly based on the supportive-accountability model of guidance in Internet interventions [51]. In this study, the primary aim of guidance will be to support participants to adhere to the training schedule. Every participant in this study group will be assigned to an eCoach during the training. The eCoaches are trained psychologists and will follow guidelines for the feedback process that are defined according to the content and structure of the intervention. At the beginning of the training, eCoachs send a message to the participants clarifying their supportive role in the program. Coaching guidance consists of two elements: a) adherence monitoring and b) feedback on demand. These principles of guidance have already been described elsewhere [35].

*Adherence monitoring* includes regularly checking whether participants have completed the intervention modules on time and sending reminders if they did not complete at least one module within 7 days. The reminders are formulated in an encouraging and motivational style to avoid reactance. In addition, all participants receive a standardized message after having completed the first module to make sure that they stick to the program.

*Feedback on demand* includes offering participants the opportunity to contact their eCoach via the internal messaging system of the training platform and to receive individual feedback whenever such a need may arise. Within 48 h, the participants will receive personalized written feedback. The time required for coaching including all reminders and feedback is estimated to be up to 1 h per participant.

#### Waiting list control (WLC)

Participants of the WLC will not get any kind of active training intervention. But they are informed that monitoring and reflecting on their drinking behavior by completing online-assessments can be a first step toward healthier drinking habits. In addition, participants will get access to the training after the 6-month follow-up assessment.

### Measures

#### Primary outcome

Primary outcome will be the self-reported alcohol consumption (standard units) during the 7 days prior to T2, using the Timeline Followback (TLFB) method [52]. The TLFB has been shown to be a valid and reliable procedure to document recent drinking histories [53]. The procedure has been proven to capture drinking levels very well compared to a daily diary [54]. It has been also validated as a web-based version [55]. Respondents retrospectively record their daily drinking by choosing the amount and kind of drinks they had out of a set of typical alcoholic drinks (e.g. a 330 ml bottle of beer or a 100 ml glass of wine). These quantities will be automatically converted to alcohol standard units and added to calculate the total sum score of units for the last 7 days.

As a secondary drinking measure, participants will be coded as responders if their drinking remains within the margin of low-risk, i.e., drinking not more than 14 (women) or 21 (men) standard units per week. Besides the drinking level, several other variables will be assessed as secondary outcomes (Table 2).

**Table 2:** Secondary outcome measures and assessment points

Outcome measures	T1	T2	T3
Alcoholic Drinks (Timeline Followback method)	✓	✓	✓
Alcohol Problems Questionnaire (APQ)	✓		✓
Readiness to Change Questionnaire (RTCQ)	✓	✓	✓
Depression Anxiety Stress Scale (DASS-21)	✓	✓	✓
Irritation Scale (IS)	✓	✓	✓
Effort Reward Imbalance Questionnaire (ERI-SF)	✓		✓
Work Limitations Questionnaire (WLQ)	✓	✓	✓
Single-Item Presenteeism Question	✓	✓	✓
Single item question on work ability	✓	✓	✓
General Self-Efficacy Scale (GSE)	✓	✓	✓
Assessment of Quality of Life (AQoL-8D)	✓	✓	✓
Trimbos and institute of Medical Technology Assessment Cost Questionnaire for Psychiatry (TiC–P-G)	✓		✓
Attitudes toward seeking psychological help (ATSPPH-SF)	✓	✓	✓
Use of other health services		✓	✓
Negative-Effects of Psychotherapy Inventory (INEP)			✓
Client Satisfaction Questionnaire (CSQ-8)		✓	

T1 = baseline assessment before randomization, T2 = post assessment after 6 weeks, T3 = follow-up assessment after 6 months

#### Secondary outcome measures

##### Alcohol-related problems

The Alcohol Problems Questionnaire (APQ) [56] will be used to measure common (23 items, e.g. “Have your friends criticized you for drinking too much?”) and occupational (8 items, e.g. „Have you been unable to arrive on time for work due to your drinking?“) alcohol-related problems. All items apply to a 6-month period prior to the assessment and can be answered by 1 = “Yes” or 0 = “No.”. Item scores can be added to calculate a common problems subdomain score (APQC) ranging from 0 to 23 and a work problems subdomain score (WORK), ranging from 0 to 8. The subdomains show internal consistencies of α = .92 for the APQC and α = .82 for WORK [57].

##### Readiness to change

The Readiness to Change Questionnaire (RTCQ) [58, 59] is based on the stage of change model of Prochaska and DiClemente. It consists of three subdomains with four items each, corresponding to the stages through which a person moves in an attempt to resolve a drinking problem: precontemplation (e.g. “I don’t think I drink too much”), contemplation (e.g. “I enjoy my drinking, but sometimes I drink too much”), and action (e.g. “I am trying to drink less than I used to”). Respondents rate all items on a five-point Likert-type scale, ranging from 1 = “strongly disagree” to 5 = “strongly agree”. The subdomains show internal consistencies of α = .82 for precontemplation, α = .86 for contemplation, and α = .78 for action.

##### Depression, anxiety, and stress

The Depression Anxiety Stress Scale (DASS-21) [60] will be used to assess symptoms of depression, anxiety, and stress with seven items each. Respondents rate each item (e.g. “I found it hard to wind down”) on a four-point Likert-type scale, ranging from 0 = “Did not apply to me at all” to 3 = “Applied to me very much, or most of the time”. Total scores of the three subdomains range from 0 to 21. The DASS-21 shows internal consistencies of α = .88 for depression, α = .82 for anxiety, and α = .90 for stress [61].

##### Work-related stress

We will use two different measures to assess work-related stress. The Irritation Scale (IS) [62] operationalizes work-related stress in terms of cognitive (CI) and emotional irritation (EI), as reactions on uncertainty in the working environment. The CI subdomain consists of three items (e.g. “Even at home I often think of my problems at work.”). The EI subdomain consists of five items (e.g. “I get grumpy when others approach me.”). Respondents rate all items on a seven-point Likert-type scale (1 = “strongly disagree”, 2 = “largely disagree”, 3 = “rather disagree”, 4 = “moderately agree”, 5 = “partly agree”, 6 = “largely agree”, 7 = “strongly agree”). The items are added to a total irritation scale. Both subdomains show good internal consistencies, ranging from α = .85 to .97 [62].

The Effort Reward Imbalance Questionnaire – Short Form (ERI-SF) [63] assesses stress based on the model of effort-reward imbalance. The subdomain “effort” consists of three items (e.g. “I have constant time pressure due to a heavy work load”). The subdomain “reward” consists of seven items (e.g. “My job promotion prospects are poor”). Respondents rate all items on a four-point Likert-type scale (1 =”strongly agree”, 2 =”agree”, 3 =”disagree”, 4 =”strongly disagree”). The subdomains show moderate to good consistencies, α = .77 for effort and α = .82 for reward [64].

##### Presenteeism

We will use the short form of the Work Limitations Questionnaire (WLQ-8) [65, 66]. It consists of eight items, measuring the degree to which health problems interfere with the ability to perform in the job. All items are to be rated on a five-point Likert-type scale, ranging from 1 =”the whole time” to 5 = “none of the time” (e.g. “In the past 2 weeks, how much of the time did your physical health or emotional problems make it difficult for you to concentrate on your work?”). In addition, we will use an adapted version of the Single-Item Presenteeism Question (“To what extent has your physical or mental health problems affected your performance at work over the past 30 days?”) [67], ranging from 0 = “not at all” to 10 “extremely”, and a single item question on work ability (“Current work ability compared with the lifetime best”) [68], ranging from 0 = “completely unable to work” to 10 “work ability at its best”.

##### Self-efficacy

The 10-item General Self-Efficacy Scale (GSE) [69] assesses a general sense of perceived self-efficacy, with the goal of predicting the ability to cope with daily problems and adapt after experiencing stressful life events. The respondents evaluate statements on a four-point Likert-type scale, ranging from 1 = „not at all true” to 4 = “completely true” (e.g. “I can typically handle whatever comes my way”). A higher score indicates higher self-efficacy. The item values can be added to a total score, ranging from 10 to 40. The internal consistency of the GSE is varying from α = .76 to .90 in different samples [69].

##### Attitudes toward seeking professional psychological help

The Attitudes Toward Seeking Professional Psychological Help Scale – Short Version (ATSPPHS-SF) [70] assesses attitudes toward seeking professional help for psychological problems. Respondents rate all of the ten items of this scale on a four-point Likert-type scale, ranging from 0 = “disagree” to 3 = “agree” (e.g. “If I believed I was having a mental breakdown, my first inclination would be to get professional attention.”). The items can be added to a total score, ranging from 0 to 30. The scale shows an internal consistency of α = .84.

##### Negative side-effects

Adverse effects will be measured with an adapted version of the Negative-Effects of Psychotherapy Inventory (INEP) [71]. The version used in this study consists of 15 items, assessing negative effects participants experienced within or after the completion of the web-based training. The INEP covers the following domains: negative intrapersonal changes, negative effects in an intimate relationship, family/friends, perceived dependence on the eCoach/intervention, and stigmatization (e.g. “I am anxious that my colleagues or friends could find out about my training participation”). Respondents rate all items on a four-point Likert scale, ranging from 0 = “no agreement at all” to 3 = “total agreement”. For each item, participants also state whether they attribute the adverse effects on the training participation or on other factors. Only item scores of those negative effects that were attributed on participating in the training are added to the total score. Higher total scores indicate more negative effects. The INEP shows an internal consistency of α = 0.85.

##### Course evaluation

To evaluate the course satisfaction we used the Client Satisfaction Questionnaire (CSQ-8) [72, 73] and adapted it to the context of web-based trainings. The CSQ consists of eight items, measuring the global client’s satisfaction with the training. Respondents rate all items (e.g. “How would you rate the quality of service you received?”) on a four-point Likert-type scale, with different responses (e.g. 1 = “Poor” to “Excellent”). Previous research indicated a high internal consistency of α = .92 for the general version [74] and α = .92 for the adapted version [75, 76].

##### Quality of life

The Assessment of Quality of Life (AQoL-8D) [77] will be used as multi-attribute utility instrument. This measure consists of 35 items, covering eight subdomains of health-related quality of life which can be combined to a physical super dimension (independent living, pain, senses) and a mental super dimension (mental health, happiness, coping, relationships, self-worth). The respondents rate all items on a four-, five-, or six-point Likert-type scale, ranging from 1 = “very rarely” to 4 = “most of the time” (e.g. “Thinking about how often you experience serious pain: I experience it…”), from 1 = “never” to 5 = “always” (e.g. “How often do you feel socially excluded or left out?”), or from 1 = “very satisfying” to 6 = “very unpleasant” (e.g. “Your close relationships (family and friends) are:”). The scale shows internal consistencies of α = .88 for the physical health dimension and α = .96 for the mental health dimension [77].

##### Cost measure

A German version of the Trimbos and Institute of Medical Technology Assessment Cost Questionnaire for Psychiatry (TiC–P-G) [78] will be used to record direct and indirect medical costs over the previous three months. Direct costs can be derived from information on the participants’ use of health services (e.g. general practice visits, sessions with psychiatrists, hospital days). To assess indirect costs participants register the number of “work loss” days (absenteeism from work) and the number of “work cut-back” days, i.e., days on that they were showing up for work despite of feeling ill (presenteeism). The questionnaire shows a good retest-reliability and achieves comparable results between patient-reported data and data derived from medical registrations [78].

### Sample size calculation

We expect that both intervention groups will be, compared to the control group, superior in terms of the primary outcome from T1 to T2. The latest meta-analysis on mainly unguided web-based interventions for reducing alcohol consumption [28] yielded an overall effect size of d = 0.20. Subgroup analyses revealed no significant differences in effect size regarding the type of intervention (personalized normative feedback vs. more extended interventions) or the number of sessions. However, in an earlier meta-analysis, the same research group found indications for extended interventions to be more effective than personalized normative feedback with an average effect size of g = 0.61 for extended and g = 0.27 for PNF [79]. Because GET.ON CWT contains evidence-based cognitive-behavioral components over and above PNF and motivational interviewing, we expect a slightly greater effect than the one that was found in the meta-analysis [28]. We aim to include 528 participants. This sample size will allow us to detect an effect size of d = 0.30 based on a power (1-ß) of 80 % and an alpha error of .05 in a two-sided test, calculated using G*Power software [80].

### Statistical analyses

The trial will be conducted in compliance with the study protocol and the Declaration of Helsinki. Aiming at an intention-to-treat design, we will include all participants who will have been randomly assigned to the conditions. Missing data will be handled using multiple imputations. In addition, per protocol analyses (PPA) will be performed, including only participants followed the intervention outlined in the study protocol. The evaluation will be conducted in accordance with the consolidated standards of reporting trials (CONSORT) [81].

### Clinical evaluation

Analyses of covariance with baseline scores as covariate will be conducted to explore the effects of the interventions compared to the control group on all primary and secondary outcomes. A-priori contrasts will be defined to test the separate effects of the guided and unguided interventions compared to the control condition. For all analyses, Cohen’s d will be calculated by subtracting the average change scores from baseline to post-assessment (T1-T2) of one study group from the other one and then dividing it by the pooled standard deviations of the change scores. We will also calculate the number needed to treat (NNT) with adherence-focused guided and unguided CWT to achieve one response, i.e., complying with the low-risk guideline, compared to the control group. For all statistical analyses, significance level will be set at p < .05 for two-sided tests.

### Economic evaluation

To compare relative costs and outcomes of the study conditions, we will conduct cost-effectiveness and cost-utility analyses from the perspectives of employers and the society. In both analyses, the incremental cost-effectiveness ratio (ICER) will be calculated for a 6-months period using the following formula: ICER = (Cost intervention group-Cost control group)/(Effect intervention group-effect control group) [82]. Treatment response will be the outcome to estimate the cost-effectiveness of the intervention whereas quality-adjusted life years (QALYs) will be the outcome to estimate cost-utility. The incremental cumulative costs will be calculated as the differences in the costs between the intervention groups and the control group. Costs to be estimated consist of direct costs for developing and maintaining the intervention, costs for staffing (i.e., for providing feedback and technical support), and opportunity costs caused by time spending on the intervention. The non-parametric bootstrap method will be used to handle uncertainty in the ICER. In addition, results will be shown in a cost-effectiveness acceptability curve [83].

### Ethical considerations

This study has been approved by the ethics committee of the Leuphana University of Lueneburg, Germany (No. Boss201404_OT).

## Discussion

Problematic alcohol consumption among the workforce is a high-risk factor for individuals in terms of disease burden, and it can lead to high costs for employers and society [1, 2, 8]. As a low-threshold health program, web-based interventions can help people displaying problematic drinking behavior [29]. However, the effects of interventions for reducing alcohol consumption are, on average, small [28, 29, 79]. While there have been a few studies on the efficacy of these kinds of interventions in the workforce [30–32], to the best of our knowledge, no research has been done on cost-effectiveness.

Based on a sample of employees from different sectors, this study provides further evidence for the (cost-)effectiveness of web-based interventions for reducing problematic alcohol consumption in the workforce. The CWT intervention draws on components of traditional methods to treat alcoholism, such as self-monitoring and reflecting on drinking behavior. In addition, techniques commonly used in cognitive-behavioral therapy and emotion regulation trainings are integral elements of the training. To the best of our knowledge, there has been no web-based intervention for reducing problematic alcohol consumption that has integrated emotional psychoeducation and emotion regulation techniques. Given that recent research provide evidence for the relevance of emotion regulation skills for abstinence from alcohol [50], integrating these techniques in interventions for problematic alcohol use may be a promising strategy to further increase the effectiveness of such interventions.

Although there are hints that guided interventions are superior compared to unguided interventions in different health problem domains [34, 84, 85], it is unclear whether this holds for interventions for reducing problematic alcohol consumption. There may be only one study directly comparing different types of guidance in a web-based intervention within the same trial [86], but there is no study on the workforce. Research on the cost-effectiveness of these interventions is also scarce. Because the operational costs of these interventions may particularly be related to the level of guidance, it is of major interest to both employers and health care providers which type of intervention is more cost-effective. The more people use an unguided intervention, the lower the costs [87]. In contrast, the costs for personnel in guided interventions are fixed and will not decrease when the number of users rises. In this three-armed trial, it will be possible to explore both the clinical effectiveness and cost-effectiveness of guided and unguided interventions.

This study has the following limitations: First, we chose a recruiting strategy that is based on the occupational health programs of several health insurance companies and did not focus on specific industrial sectors (e.g. finance, social services, health care, manufacturing, retail, or government) or specific occupational groups (e.g. managers or blue-collar workers). On the one hand, this allows us to estimate the mean (cost)-effectiveness of the intervention for the workforce. On the other hand, this certainly reduces the internal validity of the study because we cannot determine the (cost)-effectiveness for specific industrial sectors or occupational groups. Moreover, employees need to apply actively for study participation. Hence, the results may not generalize to non-help-seeking populations. Second, the economic evaluation to be conducted in this study will be based on actual intervention costs (direct intervention costs + opportunity costs for the participants). Costs associated with the implementation of the intervention (e.g. marketing) will not be considered. Third, attrition is often a problem in web-based interventions [88, 89]. Although we developed the intervention in a way that we hope keeps participants on track (e.g. they engage with integrated multimedia tools, keep an online-diary, and learn about individuals who successfully navigated exemplary situations), we expect several participants to stop using the intervention. We also expect participants to drop out of the study, i.e., to fail to take part in the follow-up assessments. Fourth, the primary aim of guidance in this study is to support participants to adhere to the intervention schedule. However, it may be the case that the level of support may be too low to have a meaningful incremental impact on the guided intervention in terms of psychopathological outcomes and cost-effectiveness compared to the unguided intervention. Fifth, due to limitations with regard to feasibility, only self-reported measurements will be used. Sixth, even though most of the self-rated measures show good psychometric properties, only a few have been validated in the context of online-assessments, for example the TLFB [55]. Seventh, in this trial, we will use a waiting list control group. This may increase the risk of overestimating intervention effects compared to an assessment-only control group [28]. However, due to practical and ethical reasons, we decided to give all control participants access to the unguided intervention after they will have finished the follow-up assessment.

## Conclusions

This study allows us to assess the (cost-)effectiveness of a web-based intervention for reducing alcohol consumption in a heterogeneous workforce. If shown effective, the CWT intervention would be a flexible solution for employees who do not use traditional services for alcohol treatment and for companies and society to overcome the high risk of ill-health and productivity losses due to alcohol-related problems. If the intervention works as intended, the next step would be to investigate which guidance format is the most feasible for dissemination to a broad community.

## Abbreviations

AUD: Alcohol use disorder
PNF: Personalized normative feedback
CWT: Clever weniger trinken (name of the intervention)
RCT: Randomized controlled trial
WLC: Waiting list control group
AUDIT: Alcohol use disorders identification test
BDI: Beck depression inventory
HAPA: Health action process approach
PST: Problem solving therapy
ART: Affect regulation training
TLFB: Timeline follow back
APQ: Alcohol problems questionnaire
RTCQ: Readiness to change questionnaire
DASS-21: Depression anxiety stress scale (short form)
IS: Irritation scale
ERI-SF: Effort reward imbalance questionnaire (short form)
WLQ-8: Work limitations questionnaire
GSE: General self-efficacy scale
ATSPPHS-SF: Attitudes toward seeking professional psychological help scale (short form)
INEP: Negative-effects of psychotherapy inventory
CSQ-8: Client satisfaction questionnaire
AQoL-8D: Assessment of quality of life
TiC–P-G: Trimbos and institute of medical technology assessment cost questionnaire for psychiatry
PPA: Per protocol analyses
CONSORT: Consolidated standards of reporting trials
NNT: Number needed to treat
ICER: Incremental cost-effectiveness ratio
QALYs: Quality-adjusted life years
